# Di-*n*-butyl­bis­(*N*-ethyl-*N*-phenyl­dithio­carbamato-κ*S*)tin(IV)

**DOI:** 10.1107/S160053681105392X

**Published:** 2011-12-23

**Authors:** Nurul Farahana Kamaludin, Ibrahim Baba, Normah Awang, Mohamed Ibrahim Mohamed Tahir, Edward R. T. Tiekink

**Affiliations:** a Environmental Health Programme, Faculty of Allied Health Sciences, Universiti Kebangsaan Malaysia, Jalan Raja Muda Aziz, 50300 Kuala Lumpur, Malaysia; b School of Chemical Sciences and Food Technology, Faculty of Science and Technology, Universiti Kebangsaan Malaysia, 43600 Bangi, Malaysia; c Department of Chemistry, Universiti Putra Malaysia, 43400 Serdang, Malaysia; d Department of Chemistry, University of Malaya, 50603 Kuala Lumpur, Malaysia

## Abstract

The title compound, [Sn(C_4_H_9_)_2_(C_9_H_10_NS_2_)_2_], features a tetra­hedrally coordinated Sn^IV^ atom; the dithio­carbamate ligands coordinate in a monodentate fashion, accompanied by two *n*-butyl chains. The non-coordinating thione S atoms are each proximate to the Sn^IV^ atom [3.0136 (7) and 2.9865 (8) Å], giving rise to distortions from the ideal geometry as evident in the wide C—Sn—C bond angle of 139.06 (12) °. In the crystal, C—H⋯S inter­actions lead to the formation of a linear supra­molecular chain along the *b* axis. The chains are aligned into layers by C—H⋯π inter­actions, and the layers stack along [001]. One of the ethyl groups is statistically disordered over two sets of sites.

## Related literature

For a review on the applications and structural chemistry of tin dithio­carbamates, see: Tiekink (2008 ▶). For related structures, see: Awang *et al.* (2010 ▶); Kamaludin *et al.* (2012 ▶).
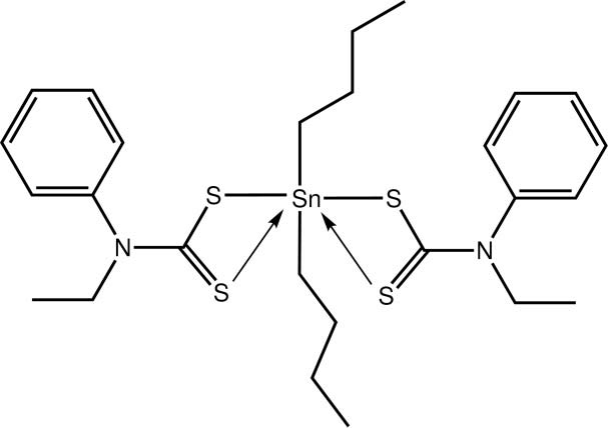


## Experimental

### 

#### Crystal data

[Sn(C_4_H_9_)_2_(C_9_H_10_NS_2_)_2_]
*M* *_r_* = 625.51
Monoclinic, 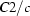

*a* = 23.9107 (7) Å
*b* = 11.9395 (4) Å
*c* = 22.0117 (7) Å
β = 106.766 (3)°
*V* = 6016.8 (3) Å^3^
*Z* = 8
Mo *K*α radiation
μ = 1.14 mm^−1^
*T* = 150 K
0.30 × 0.23 × 0.18 mm

#### Data collection

Oxford Diffraction Xcaliber Eos Gemini diffractometer
Absorption correction: multi-scan (*CrysAlis PRO*; Oxford Diffraction, 2010 ▶) *T*_min_ = 0.77, *T*_max_ = 0.81
18598 measured reflections
6072 independent reflections
5190 reflections with *I* > 2σ(*I*)
*R*_int_ = 0.028

#### Refinement

*R*[*F* ^2^ > 2σ(*F* ^2^)] = 0.030
*wR*(*F* ^2^) = 0.074
*S* = 1.03
6072 reflections
310 parameters
H-atom parameters constrained
Δρ_max_ = 0.56 e Å^−3^
Δρ_min_ = −0.48 e Å^−3^

### 

Data collection: *CrysAlis PRO* (Oxford Diffraction, 2010 ▶); cell refinement: *CrysAlis PRO*; data reduction: *CrysAlis PRO*; program(s) used to solve structure: *SHELXS97* (Sheldrick, 2008 ▶); program(s) used to refine structure: *SHELXL97* (Sheldrick, 2008 ▶); molecular graphics: *ORTEP-3* (Farrugia, 1997 ▶) and *DIAMOND* (Brandenburg, 2006 ▶); software used to prepare material for publication: *publCIF* (Westrip, 2010 ▶).

## Supplementary Material

Crystal structure: contains datablock(s) global, I. DOI: 10.1107/S160053681105392X/hb6565sup1.cif

Structure factors: contains datablock(s) I. DOI: 10.1107/S160053681105392X/hb6565Isup2.hkl

Additional supplementary materials:  crystallographic information; 3D view; checkCIF report

## Figures and Tables

**Table 1:** Selected bond lengths (Å)

Sn—S1	2.5153 (7)
Sn—S3	2.5270 (7)
Sn—C19	2.134 (2)
Sn—C23	2.143 (3)

**Table 2:** Hydrogen-bond geometry (Å, °) *Cg*1 is the centroid of the C13–C18 benzene ring.

*D*—H⋯*A*	*D*—H	H⋯*A*	*D*⋯*A*	*D*—H⋯*A*
C16—H16⋯S2^i^	0.95	2.68	3.550 (4)	152
C26—H26c⋯*Cg*1^ii^	0.98	2.85	3.810 (5)	165

Symmetry codes: (i) 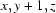; (ii) 
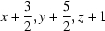
.

**Fig. 1.**
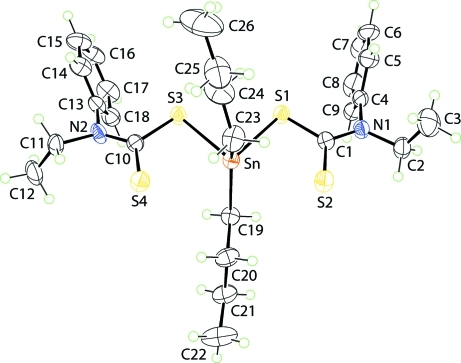
The molecular structure of (I) showing displacement ellipsoids at the 50% probability level.

**Fig. 2.**
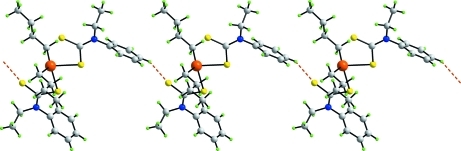
A view of the linear supramolecular chain in (I) mediated by C—H···S interactions (orange dashed lines) along the b axis.

**Fig. 3.**
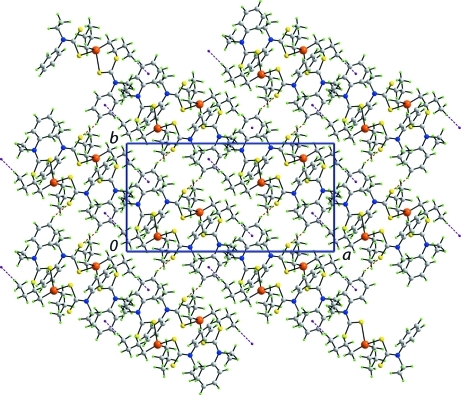
A view of the supramolecular leyer in the ab plane in (I) mediated by C—H···S and C—H···π interactions shown as orange and purple dashed lines, respectively.

**Fig. 4.**
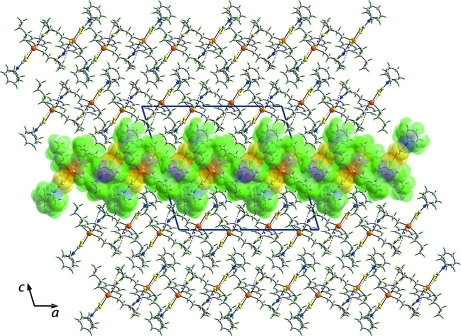
A view of the crystal packing in projection down the b axis highlighting the stacking of layers along the c axis. One layer is highlighted in space filling mode.

## supplementary crystallographic information

### Comment

The potential use of organotin dithiocarbamates as anti-cancer agents, anti-microbials and insecticides, and as synthetic precursors for tin sulfide nanoparticles, has been reviewed recently (Tiekink, 2008). In connection with recent structural studies of organotin(IV) dithiocarbamates (Awang *et al.*, 2010; Kamaludin *et al.*, 2012), the analysis of the title compound, (I), was undertaken.

The molecular structure, Fig. 1, features Sn coordinated by two dithiocarbamate ligands and two α-C atoms of the *n*-butyl groups. The dithiocarbamate ligand coordinates essentially in a monodentate fashion, an assignment supported by the large disparity in the C—S bond distances, Table 1. The coordination geometry is based on a tetrahedron with the range of tetrahedral angles being 103.55 (8) to 139.06 (12) °. The wider angle, C19—Sn—C23, is ascribed to the influence of the proximate S2 and S4 atoms [Sn···S2 = 3.0136 (7) Å and Sn···S4 = 2.9865 (8) Å].

The crystal packing of (I) features linear supramolecular chains along the *b* axis that are sustained by C—H···S interactions, Fig. 2 and Table 2. These are connected into layers in the *ab* plane by C—H···π contacts, Fig. 3 and Table 2, and the layers stack along the *c* axis, Fig. 4.

### Experimental

The title compound was prepared using an *in situ* method. A mixture of ethanol (50 ml) and *N*-ethylaniline (30 m*M*) was added to an ammonia solution (0.25%). The solution was stirred for half an hour at approximately 277 K. Carbon disulfide (30 m*M*) was added drop-wise and stirring was continued for another 6–8 h at 277 K. Di-*n*-butyltin(IV) dichloride (30 m*M*), dissolved in ethanol (20 ml), was added and stirring continued for a further 3 h. The white precipitate that formed was filtered, washed with cold ethanol and dried in a vacuum desiccator. Recrystallization as colourless prisms was from its ethanol:ethyl acetate (1:1) solution. Yield: 32%. *M*.pt. 400–401 K. Elemental analysis. Found (calculated) for C_26_H_38_N_2_S_4_Sn: C, 50.72 (49.92); H 7.47 (6.12); N 4.22 (4.48); S 20.26 (20.50) %. IR (KBr): ν(C—H) 2954 s; ν(C≐N) 1488 s; ν(N—C) 1123 m; ν(C≐S) 1004 s; ν(Sn—S) 554 s cm^-1. 13^C NMR (CDCl_3_): δ (CS_2_) 203.25 p.p.m..

### Refinement

Carbon-bound H-atoms were placed in calculated positions (C—H 0.95 to 0.99 Å) and were included in the refinement in the riding model approximation, with *U*_iso_(H) set to 1.2 to 1.5*U*_equiv_(C). The C11—C12 ethyl group was found to be disordered over two positions. The anisotropic displacement parameters for chemically equivalent atoms were constrained to be equivalent. From fractional refinement, the components were present in experimentally equivalent amounts and so were restrained to 0.5 in the final refinement.

### Crystal data

**Table:**

[Sn(C_4_H_9_)_2_(C_9_H_10_NS_2_)_2_]	*F*(000) = 2576
*M**_r_* = 625.51	*D*_x_ = 1.381 Mg m^−^^3^
Monoclinic, *C*2/*c*	Mo *K*α radiation, λ = 0.71073 Å
Hall symbol: -C 2yc	Cell parameters from 8173 reflections
*a* = 23.9107 (7) Å	θ = 2–29°
*b* = 11.9395 (4) Å	µ = 1.14 mm^−^^1^
*c* = 22.0117 (7) Å	*T* = 150 K
β = 106.766 (3)°	Prism, colourless
*V* = 6016.8 (3) Å^3^	0.30 × 0.23 × 0.18 mm
*Z* = 8	

### Data collection

**Table:**

Oxford Diffraction Xcaliber Eos Gemini diffractometer	6072 independent reflections
Radiation source: fine-focus sealed tube	5190 reflections with *I* > 2σ(*I*)
graphite	*R*_int_ = 0.028
Detector resolution: 16.1952 pixels mm^-1^	θ_max_ = 26.3°, θ_min_ = 2.2°
ω scans	*h* = −29→29
Absorption correction: multi-scan (*CrysAlis PRO*; Oxford Diffraction, 2010)	*k* = −14→13
*T*_min_ = 0.77, *T*_max_ = 0.81	*l* = −27→27
18598 measured reflections	

### Refinement

**Table:**

Refinement on *F*^2^	Primary atom site location: structure-invariant direct methods
Least-squares matrix: full	Secondary atom site location: difference Fourier map
*R*[*F*^2^ > 2σ(*F*^2^)] = 0.030	Hydrogen site location: inferred from neighbouring sites
*wR*(*F*^2^) = 0.074	H-atom parameters constrained
*S* = 1.03	*w* = 1/[σ^2^(*F*_o_^2^) + (0.0333*P*)^2^ + 5.9233*P*] where *P* = (*F*_o_^2^ + 2*F*_c_^2^)/3
6072 reflections	(Δ/σ)_max_ = 0.001
310 parameters	Δρ_max_ = 0.56 e Å^−^^3^
0 restraints	Δρ_min_ = −0.48 e Å^−^^3^

### Special details

**Table:**

Geometry. All s.u.'s (except the s.u. in the dihedral angle between two l.s. planes) are estimated using the full covariance matrix. The cell s.u.'s are taken into account individually in the estimation of s.u.'s in distances, angles and torsion angles; correlations between s.u.'s in cell parameters are only used when they are defined by crystal symmetry. An approximate (isotropic) treatment of cell s.u.'s is used for estimating s.u.'s involving l.s. planes.
Refinement. Refinement of *F*^2^ against ALL reflections. The weighted *R*-factor *wR* and goodness of fit *S* are based on *F*^2^, conventional *R*-factors *R* are based on *F*, with *F* set to zero for negative *F*^2^. The threshold expression of *F*^2^ > 2σ(*F*^2^) is used only for calculating *R*-factors(gt) *etc*. and is not relevant to the choice of reflections for refinement. *R*-factors based on *F*^2^ are statistically about twice as large as those based on *F*, and *R*- factors based on ALL data will be even larger.

### Fractional atomic coordinates and isotropic or equivalent isotropic displacement parameters (Å^2^)

**Table:**

	*x*	*y*	*z*	*U*_iso_*/*U*_eq_	Occ. (<1)
Sn	0.350914 (7)	0.637737 (15)	0.469770 (8)	0.02909 (7)	
S1	0.27097 (3)	0.68785 (6)	0.37120 (3)	0.03370 (15)	
S2	0.27694 (3)	0.44489 (6)	0.40320 (3)	0.04111 (18)	
S3	0.36947 (3)	0.84637 (6)	0.47468 (3)	0.03361 (15)	
S4	0.45001 (3)	0.71489 (6)	0.57850 (4)	0.04151 (17)	
N1	0.19430 (9)	0.53942 (18)	0.30940 (10)	0.0347 (5)	
N2	0.44536 (10)	0.93729 (19)	0.57331 (10)	0.0393 (6)	
C1	0.24355 (11)	0.5526 (2)	0.35720 (12)	0.0312 (6)	
C2	0.16723 (13)	0.4292 (2)	0.29093 (14)	0.0440 (7)	
H2A	0.1242	0.4373	0.2790	0.053*	
H2B	0.1790	0.3780	0.3278	0.053*	
C3	0.1841 (2)	0.3785 (3)	0.23662 (18)	0.0733 (11)	
H3A	0.1695	0.4254	0.1989	0.110*	
H3B	0.1672	0.3033	0.2280	0.110*	
H3C	0.2268	0.3734	0.2474	0.110*	
C4	0.16445 (12)	0.6349 (2)	0.27415 (12)	0.0349 (6)	
C5	0.17429 (15)	0.6669 (3)	0.21794 (14)	0.0539 (8)	
H5	0.2018	0.6284	0.2019	0.065*	
C6	0.14253 (18)	0.7580 (3)	0.18510 (16)	0.0708 (12)	
H6	0.1489	0.7822	0.1465	0.085*	
C7	0.10248 (17)	0.8124 (3)	0.20787 (19)	0.0690 (11)	
H7	0.0807	0.8733	0.1849	0.083*	
C8	0.09385 (15)	0.7791 (3)	0.26361 (18)	0.0593 (9)	
H8	0.0662	0.8173	0.2795	0.071*	
C9	0.12471 (12)	0.6910 (2)	0.29703 (14)	0.0407 (7)	
H9	0.1186	0.6689	0.3361	0.049*	
C10	0.42468 (11)	0.8393 (2)	0.54634 (12)	0.0308 (6)	
C11A	0.5068 (3)	0.9442 (6)	0.6239 (3)	0.0398 (12)	0.50
H11A	0.5256	0.8695	0.6303	0.048*	0.50
H11B	0.5325	0.9965	0.6094	0.048*	0.50
C12A	0.4972 (3)	0.9855 (7)	0.6843 (3)	0.0581 (13)	0.50
H12A	0.4746	1.0551	0.6761	0.087*	0.50
H12B	0.5350	0.9994	0.7157	0.087*	0.50
H12C	0.4757	0.9288	0.7008	0.087*	0.50
C11B	0.4828 (3)	0.9435 (6)	0.6395 (3)	0.0398 (12)	0.50
H11C	0.4851	0.8692	0.6600	0.048*	0.50
H11D	0.4663	0.9975	0.6638	0.048*	0.50
C12B	0.5423 (3)	0.9804 (7)	0.6389 (3)	0.0581 (13)	0.50
H12D	0.5579	0.9276	0.6138	0.087*	0.50
H12E	0.5682	0.9828	0.6824	0.087*	0.50
H12F	0.5399	1.0552	0.6199	0.087*	0.50
C13	0.41974 (12)	1.0434 (2)	0.54745 (11)	0.0353 (6)	
C14	0.44872 (14)	1.1138 (2)	0.51730 (13)	0.0445 (7)	
H14	0.4855	1.0926	0.5125	0.053*	
C15	0.42409 (16)	1.2157 (3)	0.49397 (14)	0.0520 (8)	
H15	0.4438	1.2643	0.4728	0.062*	
C16	0.37155 (16)	1.2461 (3)	0.50135 (15)	0.0560 (9)	
H16	0.3551	1.3166	0.4858	0.067*	
C17	0.34203 (15)	1.1760 (3)	0.53109 (15)	0.0536 (8)	
H17	0.3053	1.1979	0.5357	0.064*	
C18	0.36608 (13)	1.0733 (2)	0.55440 (13)	0.0426 (7)	
H18	0.3459	1.0242	0.5748	0.051*	
C19	0.30720 (12)	0.6108 (2)	0.54046 (12)	0.0353 (6)	
H19A	0.3093	0.6809	0.5651	0.042*	
H19B	0.2655	0.5960	0.5188	0.042*	
C20	0.33049 (14)	0.5158 (2)	0.58677 (14)	0.0453 (7)	
H20A	0.3726	0.5282	0.6076	0.054*	
H20B	0.3264	0.4445	0.5630	0.054*	
C21	0.29889 (14)	0.5056 (3)	0.63748 (15)	0.0522 (8)	
H21A	0.3012	0.5782	0.6598	0.063*	
H21B	0.2571	0.4893	0.6167	0.063*	
C22	0.3237 (2)	0.4151 (4)	0.6857 (2)	0.0867 (14)	
H22A	0.3192	0.3421	0.6644	0.130*	
H22B	0.3029	0.4147	0.7180	0.130*	
H22C	0.3653	0.4297	0.7059	0.130*	
C23	0.41798 (13)	0.5619 (3)	0.43672 (15)	0.0491 (8)	
H23A	0.4500	0.5367	0.4737	0.059*	
H23B	0.4018	0.4950	0.4111	0.059*	
C24	0.44233 (16)	0.6405 (3)	0.39730 (17)	0.0667 (11)	
H24A	0.4103	0.6625	0.3595	0.080*	
H24B	0.4561	0.7092	0.4224	0.080*	
C25	0.49163 (16)	0.5944 (4)	0.37556 (19)	0.0742 (11)	
H25A	0.4779	0.5272	0.3491	0.089*	
H25B	0.5236	0.5711	0.4130	0.089*	
C26	0.5151 (2)	0.6791 (6)	0.3374 (2)	0.118 (2)	
H26A	0.4824	0.7193	0.3084	0.177*	
H26B	0.5374	0.6400	0.3129	0.177*	
H26C	0.5405	0.7326	0.3664	0.177*	

### Atomic displacement parameters (Å^2^)

**Table:**

	*U*^11^	*U*^22^	*U*^33^	*U*^12^	*U*^13^	*U*^23^
Sn	0.02392 (10)	0.02876 (11)	0.03314 (10)	0.00316 (7)	0.00594 (7)	0.00012 (7)
S1	0.0307 (3)	0.0300 (4)	0.0355 (3)	−0.0002 (3)	0.0018 (3)	0.0018 (3)
S2	0.0370 (4)	0.0293 (4)	0.0491 (4)	0.0066 (3)	−0.0001 (3)	0.0014 (3)
S3	0.0316 (3)	0.0305 (4)	0.0322 (3)	−0.0003 (3)	−0.0012 (3)	0.0019 (3)
S4	0.0348 (4)	0.0326 (4)	0.0481 (4)	0.0046 (3)	−0.0025 (3)	0.0080 (3)
N1	0.0340 (12)	0.0319 (12)	0.0335 (11)	−0.0010 (10)	0.0022 (10)	−0.0010 (9)
N2	0.0438 (14)	0.0307 (13)	0.0316 (11)	0.0046 (11)	−0.0078 (10)	−0.0007 (9)
C1	0.0284 (13)	0.0325 (15)	0.0312 (13)	0.0031 (11)	0.0065 (11)	−0.0019 (11)
C2	0.0418 (16)	0.0386 (17)	0.0447 (16)	−0.0045 (14)	0.0017 (13)	−0.0042 (13)
C3	0.096 (3)	0.058 (2)	0.067 (2)	0.000 (2)	0.024 (2)	−0.0200 (18)
C4	0.0313 (14)	0.0354 (15)	0.0307 (13)	−0.0058 (12)	−0.0027 (11)	0.0046 (11)
C5	0.0498 (19)	0.068 (2)	0.0412 (17)	−0.0155 (17)	0.0085 (14)	0.0062 (15)
C6	0.075 (3)	0.080 (3)	0.0427 (19)	−0.034 (2)	−0.0072 (18)	0.0293 (18)
C7	0.060 (2)	0.048 (2)	0.075 (3)	−0.0078 (19)	−0.018 (2)	0.0238 (19)
C8	0.0477 (19)	0.0428 (19)	0.073 (2)	0.0052 (16)	−0.0060 (17)	0.0085 (17)
C9	0.0377 (15)	0.0351 (16)	0.0432 (16)	−0.0002 (13)	0.0021 (13)	0.0061 (13)
C10	0.0267 (13)	0.0324 (15)	0.0316 (13)	0.0022 (11)	0.0056 (11)	0.0020 (11)
C11A	0.030 (4)	0.037 (2)	0.043 (3)	−0.001 (3)	−0.004 (2)	0.000 (2)
C12A	0.040 (3)	0.082 (4)	0.041 (2)	−0.009 (3)	−0.0066 (19)	−0.006 (2)
C11B	0.030 (4)	0.037 (2)	0.043 (3)	−0.001 (3)	−0.004 (2)	0.000 (2)
C12B	0.040 (3)	0.082 (4)	0.041 (2)	−0.009 (3)	−0.0066 (19)	−0.006 (2)
C13	0.0468 (16)	0.0295 (15)	0.0250 (12)	0.0067 (13)	0.0031 (12)	−0.0014 (10)
C14	0.0568 (19)	0.0410 (17)	0.0368 (15)	0.0125 (15)	0.0151 (14)	−0.0006 (12)
C15	0.076 (2)	0.0409 (18)	0.0442 (17)	0.0133 (17)	0.0249 (16)	0.0091 (14)
C16	0.081 (2)	0.0412 (19)	0.0437 (18)	0.0255 (18)	0.0145 (17)	0.0128 (14)
C17	0.054 (2)	0.055 (2)	0.0503 (18)	0.0207 (17)	0.0128 (16)	0.0012 (16)
C18	0.0473 (17)	0.0386 (17)	0.0381 (15)	0.0022 (14)	0.0064 (13)	0.0009 (12)
C19	0.0322 (14)	0.0377 (16)	0.0382 (14)	0.0024 (12)	0.0138 (12)	−0.0016 (12)
C20	0.0505 (18)	0.0354 (17)	0.0568 (18)	0.0063 (14)	0.0265 (15)	0.0070 (13)
C21	0.0510 (19)	0.052 (2)	0.0594 (19)	0.0011 (16)	0.0261 (16)	0.0120 (15)
C22	0.099 (3)	0.079 (3)	0.098 (3)	0.015 (3)	0.054 (3)	0.044 (3)
C23	0.0339 (15)	0.052 (2)	0.065 (2)	0.0051 (14)	0.0197 (15)	−0.0096 (15)
C24	0.053 (2)	0.095 (3)	0.059 (2)	0.019 (2)	0.0271 (18)	0.0022 (19)
C25	0.053 (2)	0.105 (3)	0.071 (2)	−0.002 (2)	0.028 (2)	−0.026 (2)
C26	0.094 (4)	0.199 (6)	0.078 (3)	0.025 (4)	0.054 (3)	0.034 (4)

### Geometric parameters (Å, °)

**Table:**

Sn—S1	2.5153 (7)	C11B—H11D	0.9900
Sn—S3	2.5270 (7)	C12B—H12D	0.9800
Sn—C19	2.134 (2)	C12B—H12E	0.9800
Sn—C23	2.143 (3)	C12B—H12F	0.9800
S1—C1	1.736 (3)	C13—C14	1.376 (4)
S2—C1	1.689 (3)	C13—C18	1.382 (4)
S3—C10	1.743 (3)	C14—C15	1.384 (4)
S4—C10	1.682 (3)	C14—H14	0.9500
N1—C1	1.343 (3)	C15—C16	1.362 (5)
N1—C4	1.446 (3)	C15—H15	0.9500
N1—C2	1.471 (3)	C16—C17	1.376 (5)
N2—C10	1.340 (3)	C16—H16	0.9500
N2—C13	1.450 (3)	C17—C18	1.389 (4)
N2—C11B	1.476 (7)	C17—H17	0.9500
N2—C11A	1.569 (7)	C18—H18	0.9500
C2—C3	1.496 (4)	C19—C20	1.519 (4)
C2—H2A	0.9900	C19—H19A	0.9900
C2—H2B	0.9900	C19—H19B	0.9900
C3—H3A	0.9800	C20—C21	1.523 (4)
C3—H3B	0.9800	C20—H20A	0.9900
C3—H3C	0.9800	C20—H20B	0.9900
C4—C9	1.372 (4)	C21—C22	1.511 (5)
C4—C5	1.379 (4)	C21—H21A	0.9900
C5—C6	1.402 (5)	C21—H21B	0.9900
C5—H5	0.9500	C22—H22A	0.9800
C6—C7	1.367 (6)	C22—H22B	0.9800
C6—H6	0.9500	C22—H22C	0.9800
C7—C8	1.361 (5)	C23—C24	1.505 (5)
C7—H7	0.9500	C23—H23A	0.9900
C8—C9	1.371 (4)	C23—H23B	0.9900
C8—H8	0.9500	C24—C25	1.499 (4)
C9—H9	0.9500	C24—H24A	0.9900
C11A—C12A	1.499 (9)	C24—H24B	0.9900
C11A—H11A	0.9900	C25—C26	1.521 (6)
C11A—H11B	0.9900	C25—H25A	0.9900
C12A—H12A	0.9800	C25—H25B	0.9900
C12A—H12B	0.9800	C26—H26A	0.9800
C12A—H12C	0.9800	C26—H26B	0.9800
C11B—C12B	1.493 (9)	C26—H26C	0.9800
C11B—H11C	0.9900		
			
C19—Sn—C23	139.06 (12)	H12D—C12B—H12F	109.5
C19—Sn—S1	104.75 (8)	H12E—C12B—H12F	109.5
C23—Sn—S1	105.34 (9)	C14—C13—C18	120.5 (3)
C19—Sn—S3	103.55 (8)	C14—C13—N2	120.6 (3)
C23—Sn—S3	106.96 (9)	C18—C13—N2	118.9 (3)
S1—Sn—S3	83.27 (2)	C13—C14—C15	119.8 (3)
C1—S1—Sn	94.94 (9)	C13—C14—H14	120.1
C10—S3—Sn	93.96 (9)	C15—C14—H14	120.1
C1—N1—C4	120.9 (2)	C16—C15—C14	119.9 (3)
C1—N1—C2	122.5 (2)	C16—C15—H15	120.0
C4—N1—C2	116.6 (2)	C14—C15—H15	120.0
C10—N2—C13	121.9 (2)	C15—C16—C17	120.8 (3)
C10—N2—C11B	121.5 (3)	C15—C16—H16	119.6
C13—N2—C11B	114.3 (3)	C17—C16—H16	119.6
C10—N2—C11A	120.7 (3)	C16—C17—C18	119.9 (3)
C13—N2—C11A	115.8 (3)	C16—C17—H17	120.1
N1—C1—S2	122.4 (2)	C18—C17—H17	120.1
N1—C1—S1	116.69 (19)	C13—C18—C17	119.1 (3)
S2—C1—S1	120.86 (15)	C13—C18—H18	120.5
N1—C2—C3	112.6 (3)	C17—C18—H18	120.5
N1—C2—H2A	109.1	C20—C19—Sn	116.10 (18)
C3—C2—H2A	109.1	C20—C19—H19A	108.3
N1—C2—H2B	109.1	Sn—C19—H19A	108.3
C3—C2—H2B	109.1	C20—C19—H19B	108.3
H2A—C2—H2B	107.8	Sn—C19—H19B	108.3
C2—C3—H3A	109.5	H19A—C19—H19B	107.4
C2—C3—H3B	109.5	C19—C20—C21	112.9 (2)
H3A—C3—H3B	109.5	C19—C20—H20A	109.0
C2—C3—H3C	109.5	C21—C20—H20A	109.0
H3A—C3—H3C	109.5	C19—C20—H20B	109.0
H3B—C3—H3C	109.5	C21—C20—H20B	109.0
C9—C4—C5	120.6 (3)	H20A—C20—H20B	107.8
C9—C4—N1	118.4 (2)	C22—C21—C20	113.2 (3)
C5—C4—N1	121.0 (3)	C22—C21—H21A	108.9
C4—C5—C6	118.1 (3)	C20—C21—H21A	108.9
C4—C5—H5	120.9	C22—C21—H21B	108.9
C6—C5—H5	120.9	C20—C21—H21B	108.9
C7—C6—C5	120.8 (3)	H21A—C21—H21B	107.7
C7—C6—H6	119.6	C21—C22—H22A	109.5
C5—C6—H6	119.6	C21—C22—H22B	109.5
C8—C7—C6	119.8 (3)	H22A—C22—H22B	109.5
C8—C7—H7	120.1	C21—C22—H22C	109.5
C6—C7—H7	120.1	H22A—C22—H22C	109.5
C7—C8—C9	120.6 (4)	H22B—C22—H22C	109.5
C7—C8—H8	119.7	C24—C23—Sn	112.6 (2)
C9—C8—H8	119.7	C24—C23—H23A	109.1
C8—C9—C4	120.1 (3)	Sn—C23—H23A	109.1
C8—C9—H9	120.0	C24—C23—H23B	109.1
C4—C9—H9	120.0	Sn—C23—H23B	109.1
N2—C10—S4	122.87 (19)	H23A—C23—H23B	107.8
N2—C10—S3	116.46 (19)	C25—C24—C23	115.2 (3)
S4—C10—S3	120.68 (16)	C25—C24—H24A	108.5
C12A—C11A—N2	107.1 (5)	C23—C24—H24A	108.5
C12A—C11A—H11A	110.3	C25—C24—H24B	108.5
N2—C11A—H11A	110.3	C23—C24—H24B	108.5
C12A—C11A—H11B	110.3	H24A—C24—H24B	107.5
N2—C11A—H11B	110.3	C24—C25—C26	112.4 (4)
H11A—C11A—H11B	108.5	C24—C25—H25A	109.1
N2—C11B—C12B	108.3 (5)	C26—C25—H25A	109.1
N2—C11B—H11C	110.0	C24—C25—H25B	109.1
C12B—C11B—H11C	110.0	C26—C25—H25B	109.1
N2—C11B—H11D	110.0	H25A—C25—H25B	107.9
C12B—C11B—H11D	110.0	C25—C26—H26A	109.5
H11C—C11B—H11D	108.4	C25—C26—H26B	109.5
C11B—C12B—H12D	109.5	H26A—C26—H26B	109.5
C11B—C12B—H12E	109.5	C25—C26—H26C	109.5
H12D—C12B—H12E	109.5	H26A—C26—H26C	109.5
C11B—C12B—H12F	109.5	H26B—C26—H26C	109.5
			
C19—Sn—S1—C1	−73.60 (11)	Sn—S3—C10—N2	−172.91 (19)
C23—Sn—S1—C1	78.31 (12)	Sn—S3—C10—S4	7.43 (16)
S3—Sn—S1—C1	−175.89 (9)	C10—N2—C11A—C12A	−119.0 (5)
C19—Sn—S3—C10	73.17 (12)	C13—N2—C11A—C12A	75.0 (6)
C23—Sn—S3—C10	−79.19 (13)	C11B—N2—C11A—C12A	−18.7 (8)
S1—Sn—S3—C10	176.77 (9)	C10—N2—C11B—C12B	113.3 (5)
C4—N1—C1—S2	175.18 (19)	C13—N2—C11B—C12B	−83.7 (6)
C2—N1—C1—S2	−3.5 (4)	C11A—N2—C11B—C12B	16.1 (8)
C4—N1—C1—S1	−3.3 (3)	C10—N2—C13—C14	−107.8 (3)
C2—N1—C1—S1	178.0 (2)	C11B—N2—C13—C14	89.2 (4)
Sn—S1—C1—N1	174.20 (19)	C11A—N2—C13—C14	57.9 (4)
Sn—S1—C1—S2	−4.30 (16)	C10—N2—C13—C18	73.1 (3)
C1—N1—C2—C3	−96.6 (3)	C11B—N2—C13—C18	−89.8 (4)
C4—N1—C2—C3	84.7 (3)	C11A—N2—C13—C18	−121.1 (4)
C1—N1—C4—C9	−86.6 (3)	C18—C13—C14—C15	0.3 (4)
C2—N1—C4—C9	92.2 (3)	N2—C13—C14—C15	−178.8 (3)
C1—N1—C4—C5	95.3 (3)	C13—C14—C15—C16	0.5 (5)
C2—N1—C4—C5	−85.9 (3)	C14—C15—C16—C17	−0.9 (5)
C9—C4—C5—C6	−0.1 (4)	C15—C16—C17—C18	0.5 (5)
N1—C4—C5—C6	178.0 (3)	C14—C13—C18—C17	−0.7 (4)
C4—C5—C6—C7	−0.8 (5)	N2—C13—C18—C17	178.4 (2)
C5—C6—C7—C8	1.0 (5)	C16—C17—C18—C13	0.3 (5)
C6—C7—C8—C9	−0.4 (5)	C23—Sn—C19—C20	5.9 (3)
C7—C8—C9—C4	−0.5 (5)	S1—Sn—C19—C20	142.0 (2)
C5—C4—C9—C8	0.8 (4)	S3—Sn—C19—C20	−131.5 (2)
N1—C4—C9—C8	−177.4 (3)	Sn—C19—C20—C21	177.3 (2)
C13—N2—C10—S4	−175.0 (2)	C19—C20—C21—C22	−177.1 (3)
C11B—N2—C10—S4	−13.2 (5)	C19—Sn—C23—C24	−162.4 (2)
C11A—N2—C10—S4	19.9 (4)	S1—Sn—C23—C24	61.6 (2)
C13—N2—C10—S3	5.4 (3)	S3—Sn—C23—C24	−25.9 (3)
C11B—N2—C10—S3	167.1 (3)	Sn—C23—C24—C25	176.9 (3)
C11A—N2—C10—S3	−159.7 (3)	C23—C24—C25—C26	−178.6 (4)

### Hydrogen-bond geometry (Å, °)

**Table:**

Cg1 is the centroid of the C13–C18 benzene ring.

**Table:**

*D*—H···*A*	*D*—H	H···*A*	*D*···*A*	*D*—H···*A*
C16—H16···S2^i^	0.95	2.68	3.550 (4)	152
C26—H26c···Cg1^ii^	0.98	2.85	3.810 (5)	165

Symmetry codes: (i) *x*, *y*+1, *z*; (ii) *x*+3/2, *y*+5/2, *z*+1.
